# Dynamic and Structural Performances of a New Sailcraft Concept for Interplanetary Missions

**DOI:** 10.1155/2015/714371

**Published:** 2015-07-27

**Authors:** Alessandro Peloni, Daniele Barbera, Susanna Laurenzi, Christian Circi

**Affiliations:** ^1^School of Engineering, University of Glasgow, Glasgow G12 8QQ, UK; ^2^Faculty of Engineering, University of Strathclyde, Glasgow G1 1XW, UK; ^3^Department of Astronautic, Electrical and Energy Engineering, Sapienza University of Rome, Via Salaria 851, 00138 Rome, Italy

## Abstract

Typical square solar-sail design is characterised by a central hub with four-quadrant sails, conferring to the spacecraft the classical X-configuration. One of the critical aspects related to this architecture is due to the large deformations of both membrane and booms, which leads to a reduction of the performance of the sailcraft in terms of thrust efficiency. As a consequence, stiffer sail architecture would be desirable, taking into account that the rigidity of the system strongly affects the orbital dynamics. In this paper, we propose a new solar-sail architecture, which is more rigid than the classical X-configuration. Among the main pros and cons that the proposed configuration presents, this paper aims to show the general concept, investigating the performances from the perspectives of both structural response and attitude control. Membrane deformations, structural offset, and sail vibration frequencies are determined through finite element method, adopting a variable pretensioning scheme. In order to evaluate the manoeuvring performances of this new solar-sail concept, a 35-degree manoeuvre is studied using a feedforward and feedback controller.

## 1. Introduction

Solar sailing is a promising technology, which allows planning missions otherwise impracticable using traditional propulsion systems. Therefore, the solar-sailing concept opens up new avenues for scientific discoveries in many fields of astronautic science, from materials engineering to flight dynamics [1–5]. Due to the continuous and propellant-free thrust, solar sails are mainly studied for orbits with high Δ*v* requirements, such as Earth Pole-sitter orbits [6–8], orbits at the Earth-Moon libration points [9, 10], or new kinds of orbits around the Earth, as the Taranis orbits [11, 12]. Attitude control and optimal steering laws to improve sailcraft performances have recently been studied in several works as well [13–17].

Sailcrafts have a very large and complex structure, typically formed by four petal membranes, which are tensioned to form the square shape using deployable ultrathin composite booms [18–22]. Alternative methods for deploying and tensioning the membrane were also investigated and tested, such as in the case of IKAROS, the first-launched solar-sail demonstrator [23]. In that case, the four trapezoidal membranes are linked together using spaced strips, which facilitate the folding of the membrane. The solar sail was then deployed and kept extended in a flat shape by the centrifugal force due to the spin of the sailcraft itself. In all mentioned cases, even in the IKAROS demonstrator that did not use booms, the solar sail is visually and physically divided into four membranes and a central hub, which gives the typical X-configuration to the spacecraft.

This work proposes a new approach to the solar-sail design with a different kind of configuration, in which the classical central bus is divided into four hubs displaced at the corners of the square sail. With this configuration, the sail tensioning can be controlled more easily and the tensioning motors, if any, can be directly placed on the hubs. The membrane tensioning is an important topic, due to the formation of wrinkles or bubbles born from the tensioning or thermal load. This feature is crucial for the masking, shadowing, and thermal issue that may afflict sail performances. From an attitude control point of view, this configuration allows the attitude control thrusters, if any, to be directly mounted on the hubs rather than on the top of a flexible boom. Therefore, the thruster is more likely to be in the nominal position. This feature and the stiffer nature of the architecture itself entail the sail being flatter than in the X-configuration. Moreover, this type of architecture can be considered as the unit part of a bigger modular solar sail. On the other hand, the dislocated nature of this configuration increases the moments of inertia of the sailcraft, with a possible decrease of the attitude control efficiency.

This paper investigates the structural and the dynamics performances of the proposed novel configuration of the solar sail. To compare both the structural and the attitude control performances of this new architecture with those available in literature, a 40 m side sail has been considered for the study.

The paper is organised as follows: the new sail configuration is presented in Section 2; a finite element model is reported in Section 3 for the evaluation of maximum out-of-plane displacement, vibration frequencies, and calculation of the offset between the centre of mass and the centre of pressure. The offset value is used in Section 4 to investigate the solar-sail attitude control performances for a 35-degree deep space manoeuvre.

## 2. Solar-Sail Concept Configuration

**Figure 1:**
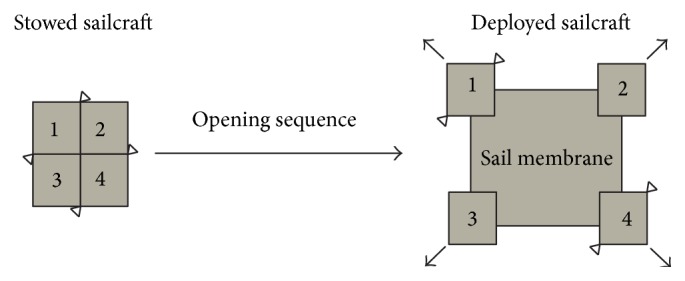
Sailcraft opening sequence.

The solar-sail geometry proposed in this study is a classical square, with the booms on the perimeter of the membrane and the mass of the satellite divided into four parts, collocated on the square's corners and joined at the booms' end. The opening sequence from the closed-shape launch configuration to the deployed one (Figure 1) is helped by the strain energy stored in the booms. The deployment velocity is a function of the booms' shapes and the parameters of the deployment mechanism [24, 25].

**Figure 2:**
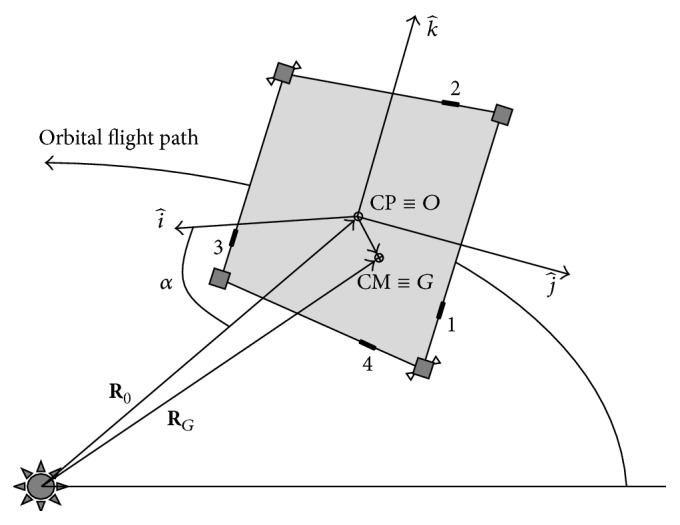
Solar sail in interplanetary trajectory.

Table 1 shows the main sailcraft's characteristics, according to [26], while Figure 2 shows the solar-sail reference frame taken into account. The sail mass in Table 1 is computed by considering the same film as in [26], in which the 1200 m^2^ sail has a mass of 6 kg. The boom mass is calculated in the same way. The origin of the body reference frame is set on the geometric centre, roll axis $\left(\hat{i}\right)$ is perpendicular to the sail plane, and pitch $\left(\hat{j}\right)$ and yaw $\left(\hat{k}\right)$ axes are the transverse axes parallel to the booms. *α* is the cone angle between the Sun-line direction and the roll axis, **R**_*G*_ is the position vector of centre of mass with respect to the Sun, and **R**_0_ is the position vector of the origin of body reference frame with respect to the Sun. The primary attitude control system is based on centre-of-mass (CM)/centre-of-pressure (CP) offset due to the shift of 4 ballast masses along the sailcraft's perimeter. The steady-state offset between the centre of mass and the centre of pressure of the proposed configuration, due to the membrane tensioning, is smaller than the one used in literature for the classical X-shaped solar sail [27–29], as will be discussed in Section 3.3. Because sliding masses do not affect rotations along roll axis, a Pulsed-Plasma-Thrusters (PPTs) system is introduced as well. In the scheme presented there are two pairs of PPTs mounted on two opposite satellites, but four pairs of thrusters can be utilized for redundancy or if greater torque on roll axis is required. It is important to underline that thrusters are mounted on a satellite at the corners of the square sail, instead of at the end of the booms as in X-configuration. This means a greater ease of assembly and a major stability of the structure. The total satellite mass considered is about 150 kg [26] and the four dislocated satellite buses have equal masses.

## 3. Structural Analysis

In this study, we performed nonlinear static analysis, based on Finite Element Method (FEM), adopting the commercial code ABAQUS. The aims of this investigation were the determination of the membrane out-of-plane deflections caused by the solar pressure, the natural modes of the structures, and the disturbing momentum due to offset between the CP and the origin of the body reference frame, varying the tension applied to the corner of the solar sail. Particular attention was paid when calculating the offset value, which will be used in the dynamics analysis to evaluate the manoeuvring performances of the novel square configuration.

The solar sail is a large thin membrane structure with the bending stiffness negligible compared to the in-plane stiffness; thus the membrane cannot carry compressive stress. The flat square shape is controlled by the use of tensioning loads applied at the membrane corners. When the tensioned membrane is exposed to the solar pressure, out-of-plane large deformations occur and wrinkles can be formed with the origin sets in the corner membrane. The numerical simulation of the wrinkles' formation is still an open issue, since the wrinkles alter the membrane shape and thus the final performances of the solar sail. Authors investigated numerically the wrinkles' amplitude tension load dependency, considering the membrane truncated at the corners in order to avoid stress concentrations in those locations [30]. However, the analysis of the wrinkles' behaviour is not the focus of this current study and will be analysed in a separate work.

In this study, we investigate the structural response of a flat, square, thin-film membrane tensioned at the corners. Considering the FEM model, the easiest way of pretensioning the membrane is to apply a tension load on the nodes positioned at the four corners. However, this approach can produce difficulties on the convergence of the numerical solution as consequence of the singularities which arise when a single force is applied to a single node of the FEM model. To avoid these singularities, Sleight and Muheim [31] proposed the use of a virtual tension obtained by applying a fictitious thermal load on the sail tensioning cable. This solution, which is a pure mathematical expedient, creates a defined displacement of the corners that correspond to a tensioning load. Furthermore, the extreme dimensions of the solar sails can add convergence problems of the nonlinear analysis. To overcome these problems, the authors in [31] used the cables, modelled as truss elements, when applying the fictitious negative temperatures and producing a membrane tensioning.

Similarly, in our FEM model, we applied a negative temperature to the cable nodes to induce the shrinkage of the wires which connect the membrane and the booms. This shrinkage strains the membrane with well-known tensioning load. In addition, the prestresses produced in the membrane help the stabilisation of the analysis and the convergence of the solution [32].

The structural simulation was composed of three steps. The first one was a linear displacement-temperature coupled step, during which a negative temperature difference was imposed on the Kevlar cables in order to prestress the membrane. The second step consisted of a nonlinear quasi-static analysis, where the load was applied linearly. In this step, the volume-proportional damping factor was considered to help the convergence of the quasi-static problem. The third step regarded the determination of the natural modes using the Subspace algorithm [33].

At the end of each simulation, the nodal translations and rotations were processed by a Matlab script to calculate the centre of pressure, which was then used to determine the offset between the centre of mass (CM) and centre of pressure (CP).

The considerations concerning the determination of the CP position started from the following equation:

(1) $$\begin{array}{c} d_{r}=\frac{1}{A_{p}}\int \left(\;\hat{s}\cdot {\hat{n}}_{a}\;\right)\rho_{a}dA, \end{array}$$

where *A*_*p*_ is the projected area of the element, $\hat{s}$ is the solar radiation unit vector, ${\hat{n}}_{a}$ is the normal to the element, and **ρ**_*a*_ is the position vector of the area element *dA*. The normal to the surface element, ${\hat{n}}_{a}$, varies as a consequence of the nodal rotation; hence an opportune rotation matrix was required to be calculated.

In our system, (1) can be rewritten in discretised form as

(2) $$\begin{array}{c} d_{r}=\overset{N}{\underset{i=1}{\sum }}\left(\;\hat{s}\cdot {\hat{n}}_{i}\;\right)\delta_{i}, \end{array}$$

where **δ** is the nodal in-plane translation. The use of triangular membrane elements avoids out-of-plane rotations and, as a consequence, the normal vector of the element is constant because of rigid deformation. The offset calculation was performed applying a solar radiation pressure (SRP) force with an angle of 35 degrees between the normal vector to the solar-sail plane and the unit vector of the solar radiation.

### 3.1. Finite Element Model

The geometry of the solar sail was schematised in three main parts: the solar-sail membrane given by a square plate with 40 m of edge; the booms represented by four large wires; the tensioning cables given by four small wires. Booms were localised at 0.25 m from the membrane edges, and the tensioning cables were positioned at the corners. The membrane flatness of the classical X-configuration is usually increased by dividing the entire sail into several strips. This helps the deployment and also reduces the stresses along the booms [34]. On the other hand, both packaging and jointing of the membrane are challenging tasks and require particular attention. The solar-sail architecture presented in this work does not require the strip strategy described above.

The material properties adopted in this work were taken from literature [31, 35] and are summarised in Table 2.

The finite element model was obtained by discretising the geometry with different element types. In particular, the sail membrane was modelled using membrane triangular elements (M3D3) with constant thickness, whereas the linear beam elements (B31) with circular cross section were used for the booms [25, 34, 36]. The tensioning cables were modelled using thermally coupled truss elements (T3D2T) as required by the displacement-temperature coupled step.

Tensioning force was calculated using a Matlab script, which implements (3) considering the Kevlar cable as an isotropic material:

(3) $$\begin{array}{c} F=T\cdot E_{\text{kevlar}}\cdot A_{\text{cable}}\cdot CTE_{\text{kevlar}}, \end{array}$$

where *A*_cable_ is the cross-section area of the cable, *E*_kevlar_ is the Kevlar Young modulus, CTE_kevlar_ is the thermal expansion coefficient, and *T* is the imposed temperature.

Assuming that the sail membrane is perfectly reflective, the total pressure load due to SRP is 2*P* = 9.12 × 10^−6^ N/m^2^. The pressure was applied in the normal direction with respect to the sail membrane. The boundary conditions were applied on the nodes at the corners of the square membrane, where a beam element and a truss element interlock. In particular, multipoint constraints (MPC) were used to connect the abovementioned nodes to a master node. In this case, the MPC type is a beam providing a rigid link between the master node and the slave ones. The master nodes may have different degrees of freedom, which can vary during the simulation. In our analysis, all master nodes were pinned during the pretensioning step, whereas the translation along the perpendicular direction with respect to the membrane plane and the relative rotation were constrained during the SRP loading step.

**Figure 3:**
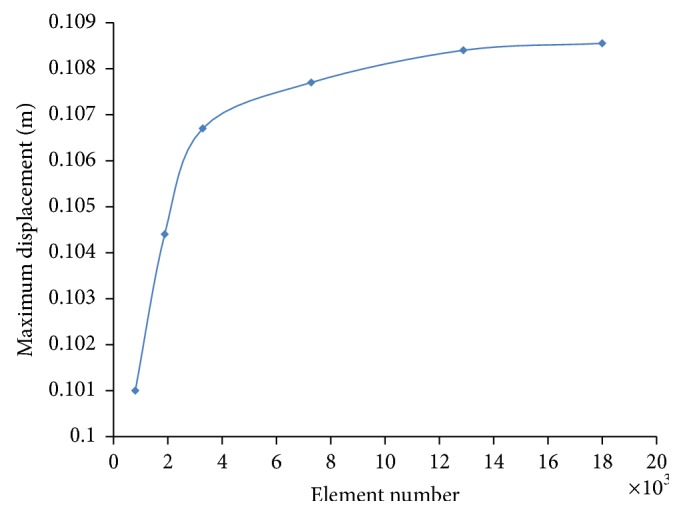
Mesh sensitivity analysis.

Before starting the simulations, a mesh convergence analysis was accomplished in order to investigate the mesh influence on both analysis and final results. In particular, we studied the effects of the number of the membrane elements, considering the maximum displacement, which occurs over the sail during the solar-pressure action as a triggering parameter. As discussed above, a pretension load is required to achieve enough stiffness, since the membrane bending rigidity is negligible. The minimum pretensioning load was calculated by a trial-and-error technique, establishing that 0.6 N for each tensioning cable is the minimum load necessary to stabilise the membrane in flat configuration.  Figure 3 shows the trend of maximum displacement of the membrane as function of the number of elements. The graph shows that over 13,000 elements the value of maximum displacement is constant; thus we adopted this number of membrane elements for the analysis.

### 3.2. Numerical Results

In this section, we present the results of the maximum out-of-plane deformation and the first three vibration frequencies due to solar radiation pressure, considering several tensioning cases. In Table 3 the results of the structural analysis for the maximum out-of-plane displacements achieved varying the tensioning force at the corners of the membrane are reported. As discussed in Section 3, the tensioning force on the membrane corners was obtained by inducing the shrinkage of the cables. This was achieved by imposing a negative temperature as boundary condition at the nodes of the truss elements. However, it is worth to note that the temperature boundary conditions reported in Table 3 are a fictitious thermal load, which is a numerical expedient to generate an effective tension applied to the membrane corners (see (3)).

**Figure 4:**
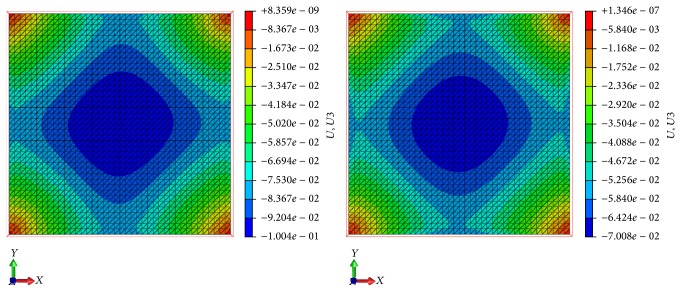
Out-plane displacement for 0.6 N and 18 N tensioning load [m].

As shown in Table 3, the first tension load at which the analysis converges is given by 0.6 N. As expected, the final out-of-plane displacement decreases with an increase of the pretensioning load.  Figure 4 shows the out-of-plane displacements obtained with the minimum tensioning force (on the left) and the maximum tensioning force (on the right), in the case of a maximum thrust during a 35-degree manoeuvre. The two displacement distributions are similar, but the maximum displacement value is largely reduced in case of tensioning at 18 N. The load required to tension the sail membrane properly is an important design key factor, because it affects the wrinkles' formation on the sail membrane [30], and it is limited by structural stability of the booms.

**Figure 5:**
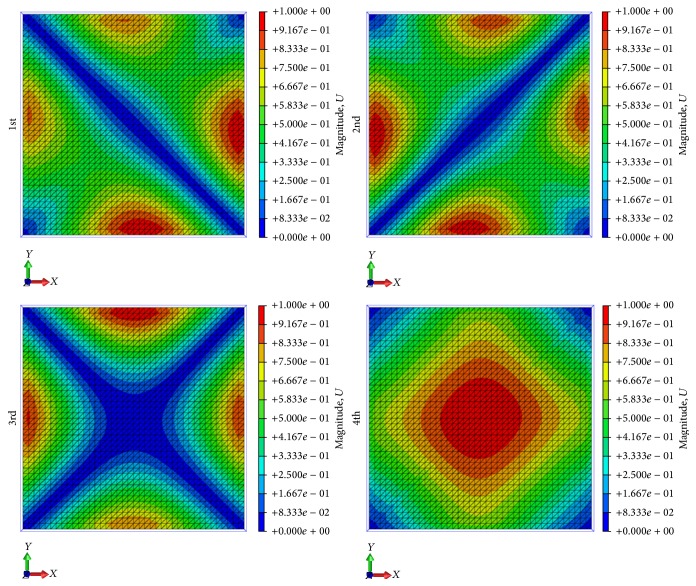
First four vibration modes for 0.6 N loading case.

**Figure 6:**
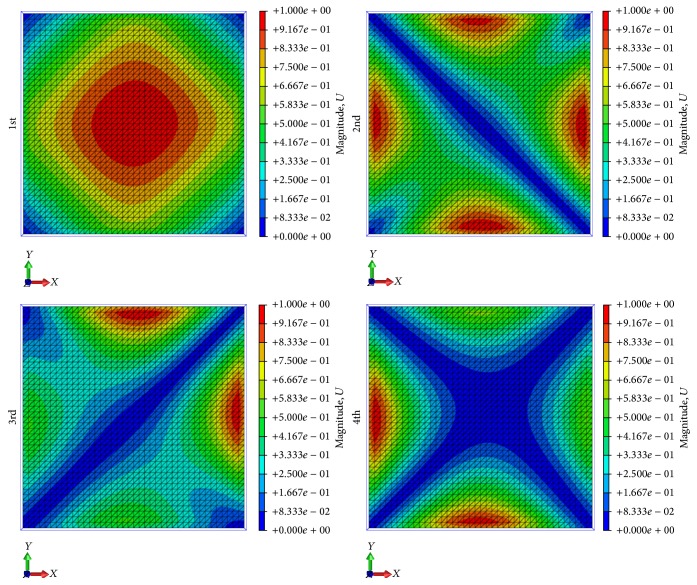
First four vibration modes for 18 N loading case.

The last part of the FEM analysis investigates the natural vibration modes of the solar-sail membrane for the different pretensioning cases. The results are reported in Table 4, where it can be observed that the vibration modes shift to higher frequencies with the increasing of the pretensioning load. These results can be related to the membrane stress state, which is directly influenced by the intensity of the tensioning load. An increase of such loads corresponds to an increase of the vibration frequency. The total displacements associated with the first four modes of the 0.6 N and 18 N tensioning cases are shown in Figures 5 and 6, respectively. Comparing the images in Figures 5 and 6, it can be observed that the shapes of the modes encountered in the 0.6 N tensioning case are different from those of the 18 N tensioning one. This difference may be explained by the loose state of the membrane in case of tension at 0.6 N. Further, the 0.6 N tension load is the lowest value of the tension force to reach the numerical convergence of the solution in the static analysis, but this value may add some uncertainties to the dynamic analysis. In fact, we noted that, in the case of pretensioning at 0.6 N, the first four modes of the solar-sail membrane presented shapes similar to ones reported in Figure 6, which are the typical shape modes of a square membrane.

### 3.3. Determination of the Structural Offset

The disturbing offset was determined using a Matlab script for each tensioning case. In this calculation, the model was simplified assuming that the booms could withstand the axial loads due to the sail tensioning and that the wrinkles of the membrane were negligible. This approach allowed us to investigate the maximum tensioning load required to reduce as much as possible the offset. The offset position on the sail plane $\left(\hat{j},\hat{k}\right)$ is greatly influenced by the SRP modelling. The solar vector is given by two components, one along the negative $\hat{i}$ direction and one along the negative $\hat{j}$ direction. Because of the symmetry of the structures, the same conclusion can be obtained considering $\hat{k}$ direction instead of $\hat{j}$ direction. Results of these calculations are reported in Table 5, where it can be noted that an increased load grants an offset reduction due to the increasing of the in-plane membrane stiffness. In particular, the solar-sail configuration proposed in this work has a smaller offset than the one for the classical X-shape configuration reported in literature [26].

The percentage change of the offsets extrapolated from the FEM analysis was taken into account for the attitude dynamics analysis. In particular, the dynamics analysis considered only the worst case offset scenario, which is the minimum applicable tensioning force (0.6 N) and is the 0.0128% of the sail edge size. The new sail concept is stiffer and guarantees a smaller offset than the X-shape one, which is 0.25% of the sail edge size [26].

## 4. Dynamics Sailcraft Performances

Due to dislocated mass, the proposed sailcraft's configuration is characterised by moments of inertia greater than the classical one. Therefore the study of the performances for an attitude manoeuvre is essential to understand whether this architecture can be a valid flight configuration. A 35-degree manoeuvre is taken into account to compare these performances with literature data. Note that, due to slow dynamics, classical interplanetary missions require small-amplitude manoeuvres per day [37, 38], while a fast manoeuvre is required only for “nonclassical” interplanetary missions [39, 40]. A body reference frame $\left\{O,\hat{i},\hat{j},\hat{k}\right\}$ is considered for modelling sailcraft attitude dynamics, as described in Section 2.

**Figure 7:**
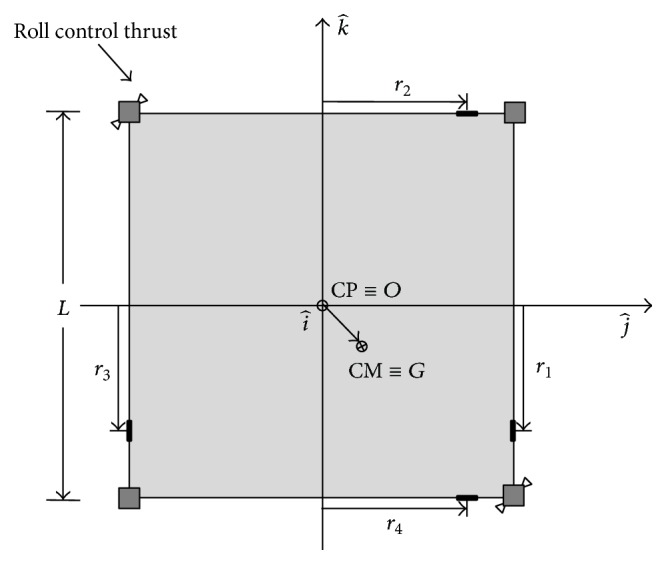
Solar-sail configuration scheme.

The primary attitude dynamics control is performed with four ballast masses (*m*_*c*_), of which masses 1 and 3 shift only along *k*-axis and masses 2 and 4 shift only along *j*-axis (Figure 7). The coordinates of the masses are reported in (4), where *L* is the sail side length and *r*_*i*_ ∈ [−*L*/2, *L*/2]

(4) r1=0L2r1,r2=0r2L2,r3=0−L2r3,r4=0r4−L2.

The sailcraft attitude dynamics is as follows [41]:

(5) $$\begin{array}{c} \frac{dh_{0}}{dt}+M\;OG\times a_{0}=T_{0}, \end{array}$$

where **h**_0_ is the angular momentum of the system referred to the origin of body reference *O*, *M* is the total sailcraft mass, **a**_0_ is the absolute acceleration of point *O*, **T**_0_ is the torque referred to *O*, and **O** **G** is the position vector of the centre of mass from the reference point *O*, as shown in

(6) $$\begin{array}{c} OG=\frac{m_{c}}{M}\overset{4}{\underset{i=1}{\sum }}r_{i}=\frac{m_{c}}{M}\left[\begin{array}{c} 0\\ r_{2}+r_{4}\\ r_{1}+r_{3} \end{array}\right]. \end{array}$$

The total angular momentum is given by the angular momentum of the bus, booms, and membrane (**h**_0,sail_) and the angular momentum of masses for attitude control (**h**_0,*m*_). In particular **h**_0,sail_ is

(7) $$\begin{array}{c} h_{0,\text{sail}}=\left[\begin{array}{ccc} J_{x} & 0 & 0\\ 0 & J_{y} & 0\\ 0 & 0 & J_{z} \end{array}\right]\cdot \left[\begin{array}{c} \omega_{x}\\ \omega_{y}\\ \omega_{z} \end{array}\right]=J_{0,\text{sail}}\cdot \omega \end{array}$$

and **h**_0,*m*_ is

(8) $$\begin{array}{c} h_{0,m}=\overset{4}{\underset{i=1}{\sum }}J_{c,i}\cdot \omega , \end{array}$$

where **J**_*c*,*i*_ is the matrix of inertia due to *i*th control mass. The **ω**-term which appears in (7)-(8) is the angular velocity vector of the spacecraft expressed as

(9) $$\begin{array}{c} \omega =\omega_{x}\hat{i}+\omega_{y}\hat{j}+\omega_{z}\hat{k}. \end{array}$$

The absolute acceleration of the reference point *O* is expressed by the absolute acceleration of the CM as

(10) $$\begin{array}{c} a_{0}=a_{G}-\frac{d^{2}OG}{dt^{2}}=\frac{F_{G}+F_{\text{SRP}}}{M}-\frac{d^{2}OG}{dt^{2}}, \end{array}$$

where **F**_*G*_ is the gravitational force vector and, considering the simplified solar-sail force model [18], the solar radiation pressure force vector is $F_{\text{SRP}}=-2\eta PA\;\cos^{2}\alpha \hat{i}$, where *P* is the value of SRP at 1 AU (*P* = 4.56 × 10^−6^ N/m^2^), *A* is the sail area, *η* = 0.85 is the solar-sail efficiency factor, and *α* is the angle between Sun-line direction and roll axis, as described in Section 2. The torque relative to the origin *O* is given by the gravitational force and SRP force, as shown in

(11) T0TG+Toff⁡≅∑i=1nmiOG+GPi·−μRGRG3−μ∇RiRi3R=RG·GPi+ε×FSRP,

where *μ* is the Sun gravitational constant, **G** **P**_*i*_ is the distance between CM and the *i*th point of the sailcraft, and **ε** is the disturbance offset vector. Since ballast masses 1, 3 and 2, 4 are coupled, (5) can be rewritten through the following scalar:

(12) 4mcr1r˙1+r2r˙2ωx+Jx+mcL2+2r12+2r22ω˙x+Jz−Jy+2mcr22−r12ωyωz−mc2M2r22r¨1+2ω˙xr2+4ωxr˙2−2ωy2r1+2ωyωzr2−2r12r¨2−2ω˙xr1−4ωxr˙1+2ωyωzr1−2ωz2r2=−3μRG3Jz−Jy+2mcr22−r12a12a13+εyFz−εzFy,4mcr1r˙1ωy+Jy+mcL22+2r12ω˙y+Jx−Jz+mcL22+2r12ωxωz−mc2M2FSRPmcr1+2r12ω˙yr1+4ωyr˙1−2ω˙zr2−4ωzr˙2+2ωxωyr2+2ωxωzr1=−3μRG3Jx−Jz+mcL22+2r12·a11a13+εzFx−εxFz,4mcr2r˙2ωz+Jz+mcL22+2r22ω˙z+Jy−Jx−mcL22+2r22ωxωy−mc2M−2FSRPmcr2−2r22ω˙yr1+4ωyr˙1−2ω˙zr2−4ωzr˙2+2ωxωyr2+2ωxωzr1=3μRG3Jy−Jx−mcL22+2r22·a11a12+εxFy−εyFx,

where (*F*_*x*_, *F*_*y*_, *F*_*z*_) are SRP force components in body reference frame and (*a*_11_, *a*_12_, *a*_13_) are the components of ${\hat{R}}_{G}$ expressed in body reference as ${\hat{R}}_{G}=a_{11}\hat{i}+a_{12}\hat{j}+a_{13}\hat{k}$ and depend on the set of rotations chosen for the attitude representation. Let us call (*φ*, *ϑ*, *ψ*), respectively, the roll, pitch, and yaw angles of the spacecraft relative to the orbital reference frame, obtained by a rotational sequence of *R*_3(*ψ*)_ − *R*_2(*ϑ*)_ − *R*_1(*φ*)_ from the orbital to the body reference frame. The kinematics equations are

(13) φ˙=1cϑcψK1+sψK2,ϑ˙=1cϑ−cϑsψK1+cϑcψK2,ψ˙=1cϑsϑcψK1+sϑsψK2+cϑK3,

where

(14) K1=ωx+cϑcψfHRGH−sφsψ+cφsϑcψHRG2,K2=ωy+cϑsψfHRGH−−sφcψ+cφsϑsψHRG2,K3=ωz−sϑfHRGH−cφcϑHRG2,fH=FSRPMi^·H^

is the force per unit mass in the orbital angular momentum direction $\left(\hat{H}\right)$. In order to achieve the desired manoeuvre, a combination of feedforward and feedback control, as described in [42, 43], is used. In Sections 4.1 and 4.2 feedforward and feedback methods are briefly presented.

### 4.1. The Feedforward Controller

Feedforward control is based on a parameterisation of a desired manoeuvre, expressed as a nth-order polynomial in the generic angle *ζ*. The order of polynomial depends on the boundary conditions and it should not be too big to reduce wandering phenomena. A seventh-order polynomial has been considered in this study as follows:

(15) ζt=ζdAτ7+Bτ6+Cτ5+Dτ4+Eτ3+Fτ2+Gτ+H,

where *ζ*_*d*_ is the desired angle of manoeuvre and *τ* = *t*/*T*_MAN_ is the nondimensional time. *T*_MAN_ is the final time after the manoeuvre. In order to find the coefficients (*A*, *B*, *C*, *D*, *E*, *F*, *G*, *H*) in (15), the boundary conditions for *ζ*(*t*) are listed in Table 6.

According to the boundary conditions in Table 6, the parameterised manoeuvre angle is expressed by the following seventh-order polynomial:

(16) ζt=ζd−20tTMAN7+70tTMAN6−84tTMAN5+35tTMAN4.

In order to design simply the feedforward controller, the following assumptions are made [42]:

(i) Inertia matrix is diagonal and constant, not affected by the position of control masses.
(ii) SRP force is the only force acting on the sailcraft.
(iii) The centre of mass lies on sail plane $\left(\hat{j},\hat{k}\right)$.

With the hypothesis above, Euler equation is simply given by

(17) $$\begin{array}{c} J\cdot \dot{\omega }+\omega \times J\cdot \omega =T_{C}+T_{\text{offset}}, \end{array}$$

where **T**_*C*_ is the control torque:

(18) $$\begin{array}{c} T_{C}={\left[\;T_{C,x},-F_{SRP}\frac{2m_{c}}{M}z,F_{SRP}\frac{2m_{c}}{M}y\;\right]}^{T}. \end{array}$$

Using (17)-(18) and defining *β* as the angle between Euler axis and pitch axis, the feedforward control law for masses is given by

(19) yt=M2mcεy−MJzζ¨tsin⁡β2FSRPmc,zt=M2mcεz+MJyζ¨tcos⁡β2FSRPmc.

To comply with geometrical boundaries, the dynamical scaling of the manoeuvre time *T*_MAN_^*∗*^ is used [44]:

(20) $$\begin{array}{c} T_{\text{MAN}}^{*}=c\cdot T_{\text{MAN}}, \end{array}$$

where

(21) c=max⁡sgn⁡y∗y∗−M/2mcεyymax−M/2mcεysgn⁡y∗,sgn⁡z∗z∗−M/2mcεzzmax−M/2mcεzsgn⁡z∗.

*y* ^*∗*^ and *z* ^*∗*^ are the maximum shift of control masses required along the *j*-axis and *k*-axis, respectively.

### 4.2. The Feedback Controller

The pitch/yaw feedback control is based on the error between the desired manoeuvre and the one carried out by the sailcraft. The feedback control logic is in Proportional-Integral-Derivative (PID) form as below [26]:

(22) $$\begin{array}{c} u=-K_{D}\dot{e}-K_{P}e-K_{I}\int e\;dt, \end{array}$$

where *e* is the error between desired angle and real one and *K*_*D*_, *K*_*P*_, and *K*_*I*_ are the derivative, proportional, and integral gain, respectively. This control can be decoupled in each axis and gains can be determined as Single-Input-Single-Output (SISO) problem. Of course, whenever tuning a gain, the system response changes and the best gains are iteratively set.

A rate limiter and a saturation limit are added to simple PID controller, because of mechanical and geometrical boundaries. These boundaries are

(23) umax=L2,u˙max=umaxTC,

where TC = 560 s is the actuator time constant taken into account, according to the value in [26]. The roll feedback control is performed with on-off controllers that work when the tolerance on roll angle is exceeded. The controller chosen is a set of 4 PPTs positioned coupled on 2 opposite satellites and the average thrust of each PPT chosen is 150 *μ*N [45]. For this study, a required roll angle of 0 degrees with a tolerance of ±0.1 degrees is set, so that the thrusters switch on when the roll angle exceeds this value.

### 4.3. Numerical Results

Numerical simulations are carried out in order to verify the performances of the proposed configuration. As reported in previous sections, a feedforward controller was used to generate the desired manoeuvre over time and a feedback controller with PID logic was set to control the nonmodelled trends in feedforward controller. The characteristics of the sailcraft are those reported in Table 1, while velocities and accelerations of masses in (12) are considered null [26, 28]. The attitude is represented by the rotational matrix which transforms body into orbital reference frame:

(24) R^Gθ^H^=−cϑcψ−cϑsψsϑcφsψ−sφsϑcψ−cφcψ−sφsϑsψ−sφcϑsφsψ+cφsϑcψ−sφcψ+cφsϑsψcφcϑi^j^k^.

**Figure 8:**
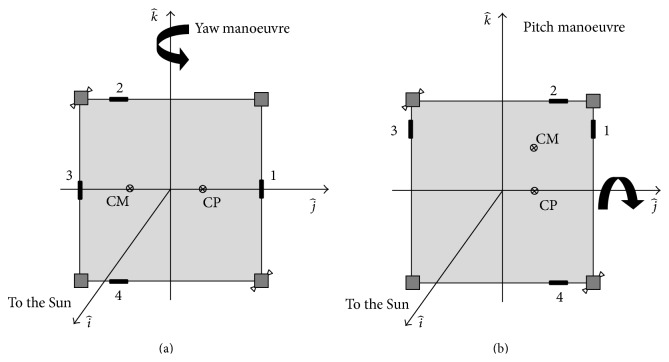
Schematisation of yaw (a) and pitch (b) manoeuvre.

In order to evaluate the performances of the proposed configuration, a 35-degree manoeuvre in a circular planar Earth-like orbit is considered. In Figure 8(a) a 35-degree yaw manoeuvre with disturbance offset on *j*-axis is schematically represented; in Figure 8(b) a 35-degree pitch manoeuvre with disturbance offset on *j*-axis is schematically represented.

**Figure 9:**
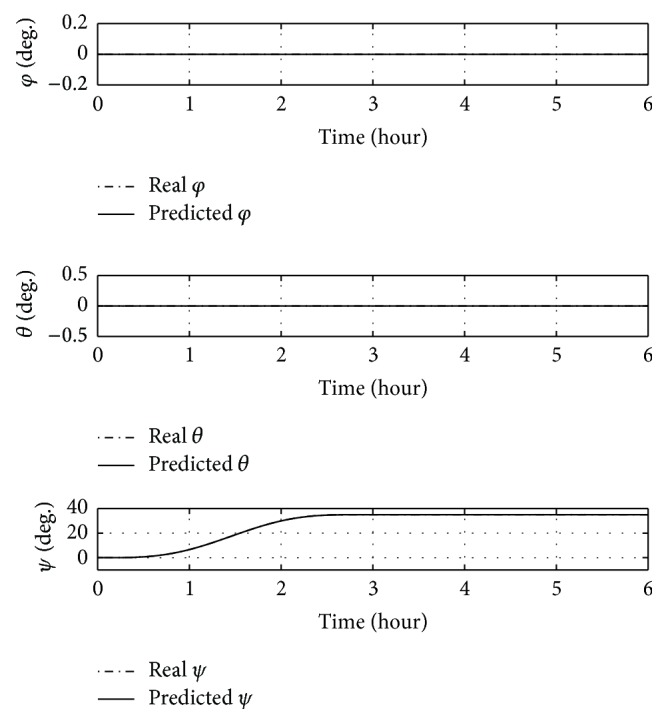
Euler angles over time for a 35-degree yaw manoeuvre without offset.

**Figure 10:**
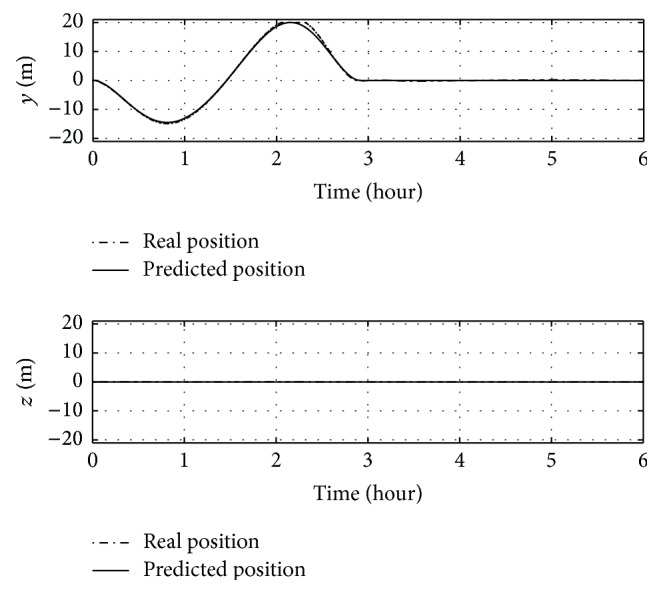
Positions of sliding masses over time for a 35-degree yaw manoeuvre without offset.

First, a 35-degree yaw manoeuvre without offset (CP ≡ *O*) is performed. Time histories of Euler angles and control masses over time are shown in Figures 9 and 10.

The manoeuvre is completed in less than 3 hours and roll and pitch angles do not change from initial conditions.  Figure 10 shows that the feedforward control ensures the success of the manoeuvre, though the real position of the sliding masses differs slightly from the predicted one, as can be seen around 2.5 hours, due to the nonmodelled forces in the feedforward controller. However, no evident differences between real angles and predicted ones are visible by Figure 9.

**Figure 11:**
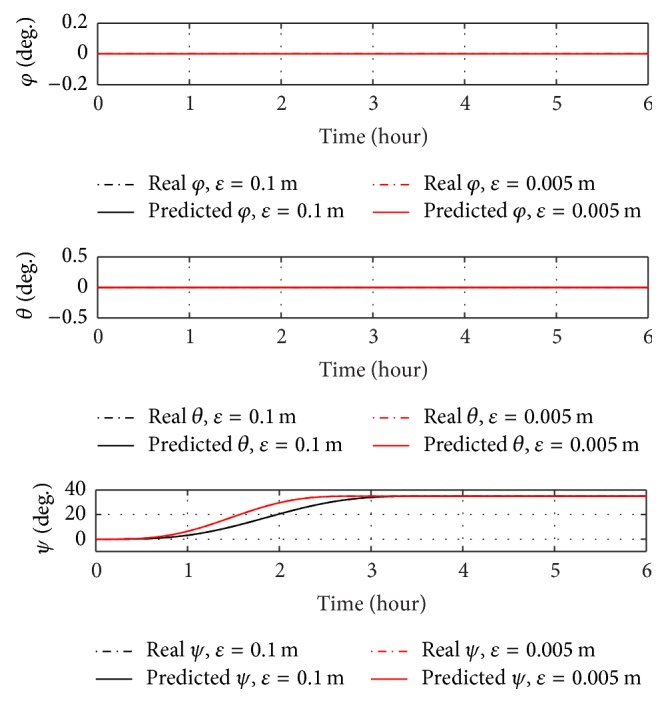
Euler angles over time for a 35-degree yaw manoeuvre with disturbance offset on *j*-axis.

**Figure 12:**
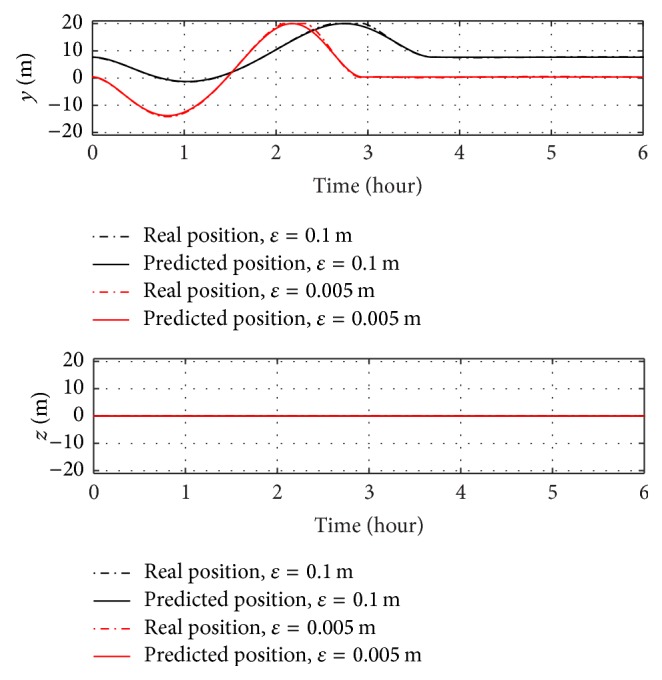
Positions of sliding masses over time for a 35-degree yaw manoeuvre with disturbance offset on *j*-axis.

The analysis with offset takes into account only the maximum offset calculated in Section 3.3, in order to have a worst-case analysis. Figures 11 and 12 show a 35-degree yaw manoeuvre with 0.005 m offset (red curve) and with the literature one of 0.1 m (black curve).

Similar to the case without offset, roll and pitch angles do not change from initial conditions and real and predicted angles overlap. The manoeuvre with the offset presented in this paper is performed in less than 3 hours, as well as in the case without offset. On the other hand, the case with the literature offset performs the manoeuvre in more than 3.5 hours. The reason for this gap can be found in the differences between the steady-state positions of control masses. The steady-state position of each sliding mass can be simply obtained by (19), as shown in

(25) ysst=M2mcεy,zsst=M2mcεz.

As shown in Figure 12 and in (25), the steady-state position of the sliding masses on *j*-axis is *y*_ss_ = 7.65  m with the literature offset of 0.1 m; on the other hand, for an offset value of 0.005 m, the steady-state position of the control masses on *j*-axis is only *y*_ss_ = 0.38 m.

**Figure 13:**
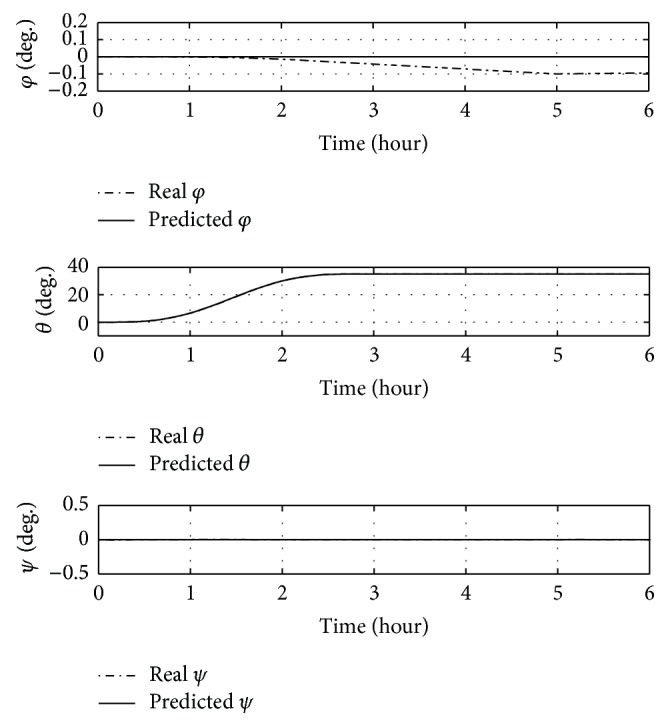
Euler angles over time for a 35-degree pitch manoeuvre with 0.005 m offset on *j*-axis.

**Figure 14:**
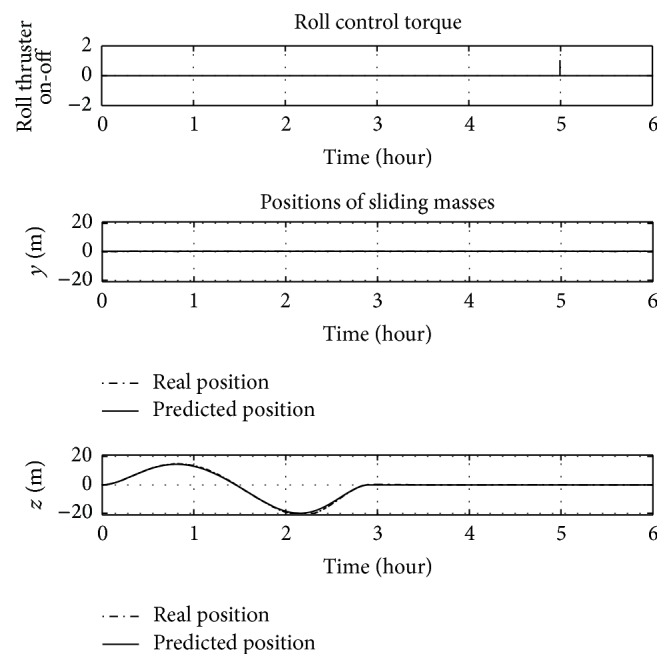
Attitude controls over time for a 35-degree pitch manoeuvre with 0.005 m offset on *j*-axis.

As shown from Figures 9 to 12, no roll control is necessary during a 35-degree yaw manoeuvre with offset on *j*-axis, because the roll angle is null during the entire manoeuvre. Figures 13 and 14 show that for a 35-degree pitch manoeuvre with a disturbance offset on the same axis a roll control is necessary.

Figure 13 shows that in a pitch manoeuvre the roll angle decreases slowly, so that only after about 5 hours does the roll controller act, due to the threshold set to 0.1 degrees. For missions that require different attitude accuracy, this threshold can be set to different values. Excluding the time history of roll angle, the manoeuvre is completed as well as in the previous examples.

Comparing these manoeuvres with those in literature, a 35-degree yaw manoeuvre presented in [26] and a 30-degree pitch manoeuvre presented in [28] are both completed in about 2 hours. The manoeuvre presented in [42] for a perfectly reflecting solar sail is much faster than the one presented here, due to the higher moments of inertia of the proposed architecture. As mentioned above, high moments of inertia are a disadvantage for an attitude manoeuvre and one of the aims of this study is to ensure the manoeuvrability of this configuration. However, in interplanetary trajectories a fast manoeuvre is not always required and the configuration presented has performances compatible with the most challenging interplanetary missions.

## 5. Conclusions

In this work, a new solar-sailing architecture has been proposed and structural and dynamics performances have been investigated. The structural analysis has shown that the new solar sail is stiffer than the central-hub sail. This characteristic produces smaller out-of-plane deformation and, as a consequence, a reduction of the disturbing centre-of-mass/centre-of-pressure offset. Furthermore, the proposed configuration can easily manage this offset with an opportune sail tensioning load. The applied tensioning load depends on the booms' stiffness and it can be foreseen that it affects greatly the membrane wrinkling and that it increases with the load. In the proposed configuration, the tensioning motors, the sliding masses motors, and PPTs can find a good location in the corner hubs, whereas in the central-hub sail they are positioned at the end of the booms. This can allow the designing of a real shape and vibration membrane control, reducing the offset with respect to the offset reported in literature for the central-hub sail. Furthermore, the corner-tensioned configuration produces more important effects such as the reduction of the masking, the shadowing, and the local thermal problems that can be controlled during the flight. In addition, it is worth noting that in the classical X-configuration the effective area for a 40 m side sail is 1200 m^2^ instead of 1600 m^2^, due to the membrane deformation and the fact that around the booms there is no membrane. On the contrary, in the proposed configuration the disposition of the booms along the perimeter and the deformation of the membrane allow one to consider a major effective area, closer to nominal value of 1600 m^2^. Starting from these results, numerical simulations of the attitude manoeuvres demonstrated that the proposed architecture gives good performances, despite the large moments of inertia. It was shown that a 35-degree manoeuvre can be completed in less than 3 hours, according to the usual requirements for interplanetary missions. The new solar-sail concept is proposed for an interplanetary mission, but advantages of this configuration are useful also for planetary missions. In general, the principal constraint that must be taken into account is the velocity of change attitude manoeuvres. In fact, for a planetary mission, around the nodal line a fast change on the sail attitude can be necessary; this event can be critical for this configuration and the performances must be evaluated case by case. On the contrary the higher moment of inertia can be favourable for missions where the sail attitude must remain constant (but this is valid also for the interplanetary case). In all cases studied, the disturbing torque, caused by structural offset, determines the steady-state positions of the sliding masses. As a consequence, the small offset value of this sail configuration guarantees a great increase in manoeuvrability.

## Figures and Tables

**Table 1:** Sailcraft properties.

Sail side [m]	40
Sail area [m^2^]	1600
Sail mass [kg]	8
Boom mass [kg]	10
Control mass (each) [kg]	1
Satellite mass (each) [kg]	33
Sailcraft total mass [kg]	153
*J*_*x*_ (roll) [kg·m^2^]	1.1 × 10^5^
*J*_*y*_ (pitch) [kg·m^2^]	5.6 × 10^4^
*J*_*z*_ (yaw) [kg·m^2^]	5.6 × 10^4^

**Table 2:** Materials properties.

Components	Material	Radius [m]	Thickness [m]	Modulus [N/m^2^]	Poisson's ratio	Density [kg/m^3^]
Boom	Composite	0.15	0.0004	124 × 10^9^	0.30	1908
Tensioning cable	Kevlar	0.0005	N/A	62 × 10^9^	0.36	1440
Membrane	CP1	N/A	3.5 × 10^−6^	2.17 × 10^9^	0.34	1434

**Table 3:** Pretensioning scheme results.

Boundary cable temperature [°C]	Effective tensioning load [N]	Maximum out-of-plane displacement [m]
−25	0.6	0.1004
−50	1.12	0.09938
−100	2.3	0.09734
−400	9	0.08520
−800	18	0.07005

**Table 4:** Vibration frequency.

Tensioning case 0.6 N		Tensioning case 18 N	
Mode number	Frequency [Hz]	Mode number	Frequency [Hz]
1	3.01406*E* − 02	1	3.40415*E* − 02
2	3.04789*E* − 02	2	3.61394*E* − 02
3	3.11011*E* − 02	3	3.73543*E* − 02
4	3.77720*E* − 02	4	3.79401*E* − 02

**Table 5:** Offset calculation results.

Force [N]	Offset [m]	Offset [%]
0.6	0.0051	0.0128
1.12	0.005	0.0125
2.3	0.0049	0.0123
9	0.0041	0.0103
18	0.0034	0.0085

**Table 6:** Feedforward boundary conditions.

*n*	${\left.\frac{d^{n}\zeta \left(t\right)}{dt^{n}}\right|}_{t=0}$	${\left.\frac{d^{n}\zeta \left(t\right)}{dt^{n}}\right|}_{t=T_{\text{MAN}}}$
0	0	*ζ*_*d*_
1	0	0
2	0	0
3	0	0
